# SV40 Hijacks Cellular Transport, Membrane Penetration, and Disassembly Machineries to Promote Infection

**DOI:** 10.3390/v11100917

**Published:** 2019-10-05

**Authors:** Yu-Jie Chen, Xiaofang Liu, Billy Tsai

**Affiliations:** Department of Cell and Developmental Biology, University of Michigan Medical School, 109 Zina Pitcher Place, BSRB 3043, Ann Arbor, MI 48109, USA; chenyuj@med.umich.edu (Y.-J.C.); xiaofanl@med.umich.edu (X.L.)

**Keywords:** SV40, nonenveloped virus, endoplasmic reticulum, membrane penetration, viral disassembly

## Abstract

During entry, a virus must be transported through the endomembrane system of the host cell, penetrate a cellular membrane, and undergo capsid disassembly, to reach the cytosol and often the nucleus in order to cause infection. To do so requires the virus to coordinately exploit the action of cellular membrane transport, penetration, and disassembly machineries. How this is accomplished remains enigmatic for many viruses, especially for viruses belonging to the nonenveloped virus family. In this review, we present the current model describing infectious entry of the nonenveloped polyomavirus (PyV) SV40. Insights from SV40 entry are likely to provide strategies to combat PyV-induced diseases, and to illuminate cellular trafficking, membrane transport, and disassembly mechanisms.

## 1. Introduction

To cause infection, a virus must traverse through the elaborate inter-connected endomembrane system of the host cell, reaching an intracellular organelle where it escapes into the cytosol via penetration of a cellular membrane. Upon cytosol arrival, the virus is often mobilized further into the nucleus to enable viral replication. Importantly, viral disassembly is coupled to different entry steps to ensure eventual delivery of the virus to the replication site. Although this entry process remains largely mysterious for the nonenveloped virus family, a more coherent understanding is nonetheless slowly emerging [1,2,3].

The first step of nonenveloped virus entry typically requires the viral particle to engage a host receptor displayed at the cell surface [4,5,6]. Because a nonenveloped virus lacks a surrounding membrane, a structural protein on the surface of the virus is responsible for this interaction. This binding event triggers receptor-mediated endocytosis, trafficking the virus to the endosomes. This compartment can be considered the critical “sorting station,” as an endosome-localized virus is subsequently sorted along different pathways that dictate the fate of the virus. For instance, if a virus sorts to the lysosomes from the endosomes, it often experiences a non-productive fate, since proteolytic degradation of the virus in the lysosomes inactivates it. Alternatively, rather than sorting to the lysosomes, a virus may penetrate the endosome membrane, thereby escaping into the cytosol and then the nucleus to trigger infection—this productive route most aptly describes the fate of the nonenveloped adenovirus [7]. Golgi targeting from the endosomes is another pathway that can lead to successful infection. For instance, in the case of the nonenveloped human papillomavirus (HPV), the endosome-localized HPV is sorted to the Golgi apparatus where it remains hidden until mitosis. In this phase of the cell cycle, Golgi membrane fragmentation enables HPV to bud into vesicles that gain nuclear entry (due to disassembly of the nuclear membrane during mitosis) in order to promote infection [8,9,10].

Intriguingly, there exists yet another productive infection pathway that emanates from the endosomes—sorting to the endoplasmic reticulum (ER). However, in contrast to the classic retrograde transport pathway [11,12], this endosome-to-ER route bypasses the Golgi. Entry of the nonenveloped polyomavirus (PyV) family in fact exploits this endosome-to-ER pathway [13,14]. Because significant insights have recently emerged regarding PyV entry, this review will focus on clarifying the molecular and cellular basis of PyV infection. 

PyVs cause debilitating human diseases, especially in immunocompromised patients. Prominent human PyVs include the BK PyV that induces hemorrhagic cystitis and nephropathy, JC PyV that causes progressive multifocal leukoencephalopathy, and the Merkel cell PyV that triggers Merkel cell carcinoma [15,16,17]. Simian virus 40 (SV40) is the archetype PyV, possessing structural and genetic similarities to human PyVs and sharing a similar infection pathway as its human counterparts [18,19,20,21]. Not surprisingly, studies on SV40 entry have illuminated the cellular basis of human PyV infection.

**Figure 1:**
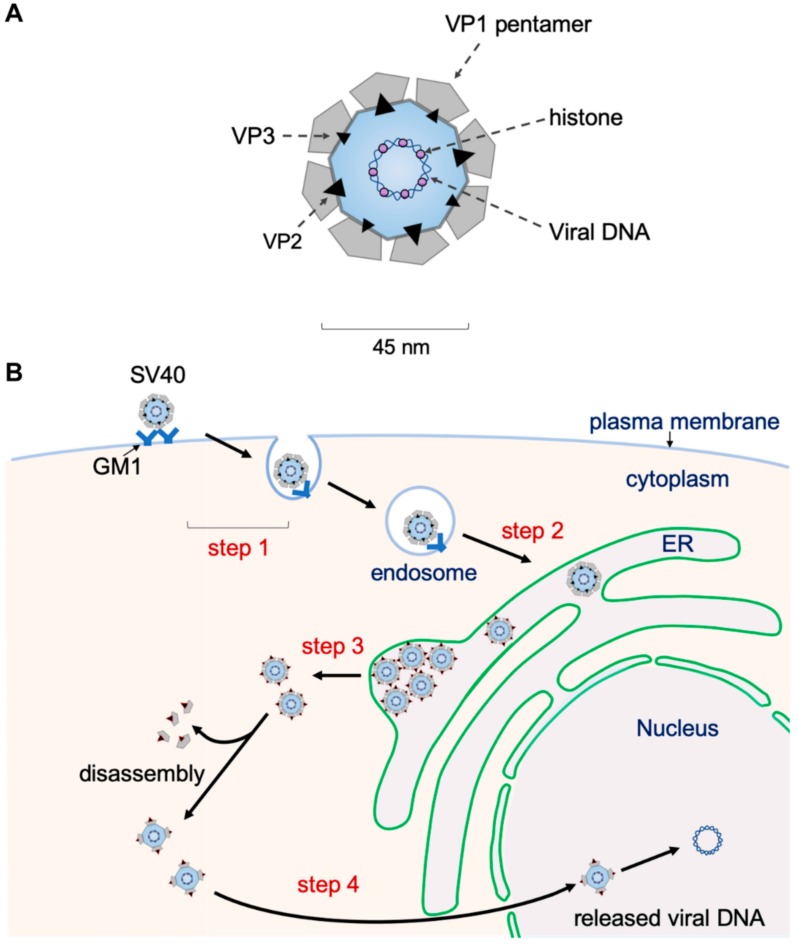
SV40 structure and entry pathway. (**A**) Diagram of the SV40 structure. (**B**) To begin the entry process, SV40 binds to a host cell receptor at the plasma membrane called ganglioside GM1 (step 1). This event initiates receptor-mediated endocytosis that targets the virus to the endosomes. From the endosomes, SV40 is targeted to the ER (step 2) from where it breaches the ER membrane to escape into the cytosol (step 3). Upon reaching the cytosol, the virus is further mobilized into the nucleus (step 4) where its genome is released. Transcription and replication of the viral genome then lead to lytic infection or cellular transformation.

Structurally, SV40 consists of 72 pentamers of the major structural protein VP1 that encases its 5 kilobase-pair double-stranded DNA genome, with each pentamer harboring an internal hydrophobic structural protein VP2 or VP3 [18,19,20] (Figure 1A). When assembled, the viral particle has as a diameter of 45 nm. To infect cells, SV40 binds to a host cell receptor at the plasma membrane, initiating receptor-mediated endocytosis that targets SV40 to the endosomes (Figure 1B, step 1). From the endosomes, the virus is sorted to the ER (step 2) from where it penetrates the ER membrane to escape into the cytosol (step 3). Upon reaching the cytosol, the virus is delivered into the nucleus (step 4) where transcription and replication of the viral genome lead to lytic infection or cellular transformation. We will detail each of these steps below. 

## 2. Receptor-Mediated Endocytosis to Endosomes

The host entry receptor for SV40 is a glycolipid molecule called ganglioside GM1 [4,22]. This virus-ganglioside interaction appears to be conserved because the murine PyV uses gangliosides GD1a and GT1b as its functional entry receptor [4,23], while the human BK PyV binds to gangliosides GD1b and GT1b to enter host cells leading to infection [6]. The use of ganglioside as an entry receptor is not exclusive to PyV, as certain bacterial toxins belonging to the so-called AB_5_ family, including cholera toxin (CT) and shiga toxin (ST), also exploit gangliosides on the plasma membrane as entry receptors to promote cellular intoxication [24,25]. One striking similarity between PyV and these AB_5_ toxins is that their receptor-binding subunits—VP1 for PyV and the B-subunit for CT/ST—form pentamers. Moreover, it has been proposed that when these pentameric structures engage their respective ganglioside receptor on the cell surface, the ganglioside can aggregate, forming a physical platform that activates an intracellular signal transduction cascade that assist in the endocytic process [24,26].

**Figure 2:**
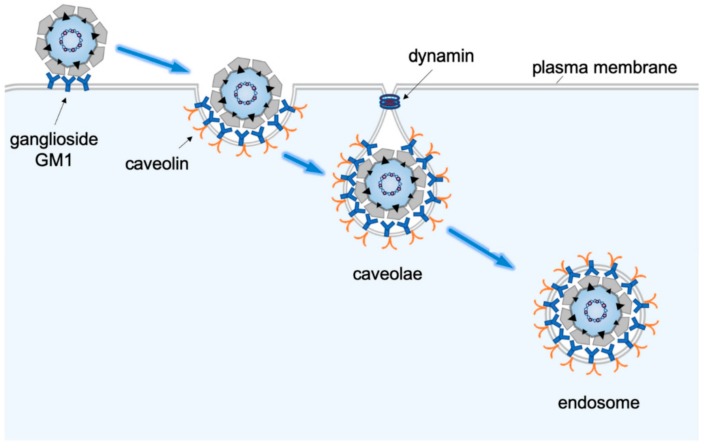
SV40 cell entry via caveolae-dependent endocytosis. The host entry receptor for SV40 is the glycolipid molecule called ganglioside GM1. Receptor-engagement enables caveolae-dependent endocytosis that targets the virus to the endosomes. From the endosomes, the virus is targeted to the ER.

Structurally, a ganglioside is an amphipathic lipid molecule containing a hydrophobic ceramide domain that inserts into the outer leaflet of the plasma membrane, and a hydrophilic carbohydrate moiety facing the extracellular space which binds directly to the virus. When VP1 of SV40 engages ganglioside GM1, the underlying membrane becomes deformed, causing deep invagination and tubulation of the plasma membrane (Figure 2 [27]). Strikingly, the presence of the long acyl chains of the ceramide domain within GM1 is required for virus-induced tubulation of the plasma membrane [27]. Such robust induction in membrane curvature enables the virus to internalize into tight-fitting vesicles, which subsequently deliver SV40 to endosomal compartments including the early and late endosomes [14,28]. Computational modeling suggests that each SV40 particle engages four receptor molecules in order to achieve stable interaction, and that the ability of the receptor to freely diffuse within the plane of the plasma membrane is essential for this steady interaction [29]. The initial internalization step is not mediated by canonical clathrin-mediated endocytosis, but instead relies largely on a caveolae-dependent entry mechanism, although other entry pathways remain possible [30,31,32,33,34,35]. SV40 in caveolae induces actin breakdown, followed by actin recruitment, events that may be coupled to the activation of cellular signaling molecules including tyrosine kinases [36]. In addition, the dynamin GTPase is also recruited to the virus-containing caveolae structure [36], presumably to execute the critical scission reaction that generates the endocytic vesicle harboring SV40 destined for the endosomal compartments.

Functionally, chemical disruption of the low endosomal pH has been shown to block SV40 [28], as well as murine PyV [28], BK PyV [37,38], and JC PyV, infections [39]. There are at least two different explanations to account for these observations. First, because disruption of the endosomal pH is known to perturb proper endosomal maturation and function [40], an intact endosomal system is likely essential for productive SV40 entry. Alternatively, a low pH may be required to directly trigger a conformational change in the viral particle to promote infection. In this regard, there is evidence that the low endosomal pH can directly impart structural changes to the murine PyV [14]. How this low pH-dependent conformational change promotes PyV infection remains unclear, but it presumably facilitates subsequent architectural rearrangements essential for successful viral infection.

## 3. Targeting to the Endoplasmic Reticulum

From the endosomes, SV40 bypasses the Golgi and is instead sorted directly to the ER. This is based on the observation that, although SV40 colocalizes with different ER markers and can be found (by electron microscopy) to be in the smooth ER during entry [13,28], the virus has not been reported to colocalize with any Golgi markers after endocytosis [28]. This entry mechanism is in stark contrast to CT and ST, which are targeted to the Golgi prior to ER arrival during cellular intoxication [24,25,26]. 

The molecular basis by which endosome-localized SV40 reaches the ER is rather obscure. In the case of the murine PyV, the ganglioside GD1a receptor that first binds to the virus at the cell surface in fact executes an important intracellular role by targeting the virus from the endosomes to the ER [4,23]. By analogy, ganglioside GM1 might also promote endosome-to-ER trafficking of SV40. In the endosomes, multimerization of the gangliosides (as a result of PyV binding) may transmit a signal across the endosome membrane to recruit cytosolic factors that couple the endosomes to the ER. For instance, the endosome-localized human BK PyV exploits the activity of the cytosolic Rab18 small GTPase to traffic from the endosomes to the ER [41].

An outstanding question is how the SV40-containing endosomes physically “dock” on the ER membrane to facilitate entry of the endosome-localized virus into the ER lumen. One possibility is that a yet-to-be identified ER membrane protein binds simultaneously to the endosomes and a component of the ER membrane’s fusion machinery. This setup tethers the endosomes to the ER and allows the endosomes (or a vesicle derived from the endosomes) to locally fuse with the ER membrane via the action of the fusion machinery. As a consequence of the fusion reaction, endosome-localized SV40 reaches the ER lumen. In this context, the ER membrane fusion component syntaxin 18 has been found to promote ER-arrival of BK PyV from endosomal compartments [41]. Because a typical membrane fusion reaction requires the contribution of a corresponding fusion component from the donor (i.e., endosome) membrane, we envision that the endosomes likely provide a fusion activity to support endosome-ER membrane fusion. 

## 4. ER Membrane Penetration to Enable Viral Escape into the Cytosol

**Figure 3:**
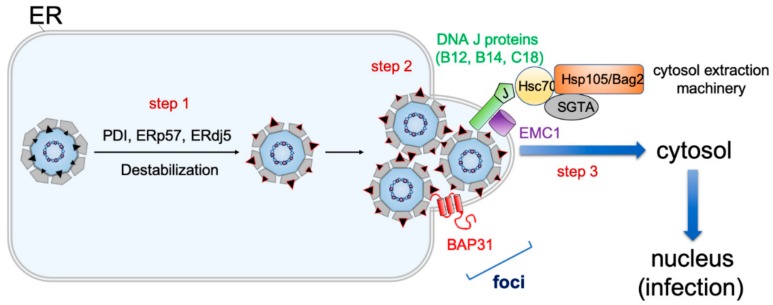
ER-to-cytosol membrane escape of SV40. To initiate ER-to-cytosol membrane escape of SV40, the ER-resident redox proteins PDI, ERp57, and ERdj5 reduce and isomerize the disulfide bonds of the virus (step 1), generating a partially destabilized hydrophobic particle that inserts into the ER membrane. In the ER membrane, EMC1 acts to stabilize the membrane-inserted virus to prevent more pronounced viral disassembly that would preclude entry into the cytosol. To reach the cytosol, SV40 induces the formation of a membrane penetration site called foci (step 2), a structure where select host components including BAP31 and DNA J proteins (B12, B14, and C18) are reorganized. The subsequent recruitment of a cytosol extraction machinery (composed of Hsc70, Hsp105, Bag2, and SGTA) to the J proteins enables the ejection of the ER membrane-inserted SV40 into the cytosol (step 3). Cytosol-localized virus finally enters the nucleus to promote infection.

Upon arrival into the ER lumen, SV40 must penetrate the ER membrane in order to escape into the cytosol. Exciting new insights illuminating this step have been revealed in recent years. To prime SV40 for ER-to-cytosol membrane penetration, ER-resident redox proteins including PDI, ERp57, and ERdj5, reduce and isomerize the disulfide bonds of the viral particle (Figure 3, step 1 [42,43,44,45,46]). Because these covalent bonds provide crucial architectural support for SV40, it is not surprising that disulfide bond reductions and isomerization destabilize the viral particle. For the murine PyV, another redox protein called ERp29 was shown to act as a chaperone to locally unfold the C-terminal arms of VP1 [44,45], which normally stabilize inter-pentamer interactions of the virus. As a consequence of these destabilizing events, the internal hydrophobic proteins VP2 and VP3 become exposed [43,47,48], generating a hydrophobic particle that binds to and integrates into the ER membrane.

To escape into the cytosol from the ER membrane, incoming SV40 remodels the ER membrane to create a penetration site (called foci) from where it enters the cytosol (Figure 3, step 2 [49,50,51,52]. During foci formation, SV40 reorganizes select ER membrane proteins into the foci structure. For example, the membrane protein BAP31 relocates into the foci, where it interacts with the aforementioned membrane-inserted SV40 to initiate membrane translocation into the cytosol [51]. Within the ER membrane, the EMC1 membrane protein acts as a transmembrane chaperone to stabilize membrane-embedded SV40, thereby preventing premature disassembly of the viral particle to ensure proper penetration [53]. SV40 also triggers the transmembrane DNA J proteins B12, B14, and C18 to accumulate in the foci—this in turn recruits a cytosol chaperone complex (composed of Hsc70, Hsp105, SGTA, and Bag2) that extracts SV40 into the cytosol to complete the escape process (Figure 3, step 3 [49,50,54,55,56]). Critical evidence supporting the notion that the virus-induced foci structure serves as SV40’s cytosol entry portal includes the observation that (1) proper foci formation is required to promote viral infection [49,52,53], (2) foci structures are formed prior to cytosol arrival of the virus [49,52,53,54,55], (3) membrane penetration-competent SV40 preferentially localizes in the foci [52,57,58], (4) selective ER membrane proteins that promote ER membrane penetration of SV40 reorganize to the foci while those that are dispensable in this transport event do not [49,50,51,52,53,59], and (5) perturbing cytosolic factors that extract SV40 from the ER into the cytosol trap the virus in the foci [49,54,55]. 

How might the SV40-induced foci be generated? We envision that foci formation likely requires significant mechanical force. To address how this is achieved, we recently discovered that the force generated by the kinesin-1 motor is in fact harnessed to build the foci structures [58]. Specifically, SV40 exploits the force of kinesin-1 in order to construct a large, mature functional focus by coalescing multiple smaller immature foci structures. However, what remains unclear is how SV40 in the ER lumen is able to transmit a signal across the ER membrane in order to activate the motor activity of the cytosol-localized kinesin-1. Equally unclear is the identity of the ER membrane cargo that directly engages kinesin-1 to promote foci generation. Indeed, these questions deserve future investigations. 

## 5. Nuclear entry

Once SV40 reaches the cytosol, it must mobilize into the nucleus to cause infection. To reach the nucleus, a cellular cargo typically transports across the nuclear pore complex (NPC) embedded in the nuclear membrane whose pore size is approximately 5–10 nm [60]. During nuclear entry, a cellular cargo uses its nuclear localization signal (NLS) to recruit importin α, a member of the heterodimeric nuclear import receptor complex which consists of importin α and importin β [61,62,63,64]. Once importin α/β delivers the cargo to the NPC, subsequent cargo interactions with various components of the NPC propel it into the nucleoplasm. 

In this context, two critical events must occur prior to nuclear entry of cytosol-localized SV40: Physical transport to the NPC and capsid disassembly to generate a smaller subviral particle that can fit through the pore of the NPC. Virus-induced destabilization of the nuclear membrane’s integrity may further facilitate the nuclear entry of SV40, as has been suggested [65]. At present, it remains unknown whether cytosol-localized SV40 is first transported to the NPC where disassembly ensues, or alternatively, whether the virus is initially disassembled in the cytosolic space and then targeted to the NPC. The former scenario offers the virus an advantage because capsid disassembly in the cytosol exposes the underlying viral genome which elicits a host immune response [66]. SV40 might be able to avoid this response entirely by undergoing disassembly immediately prior to nuclear entry. 

Regardless of the scenario, we recently reported that another host motor—cytoplasmic dynein-1 (hereafter referred to as dynein)—plays a critical role in facilitating SV40 disassembly to promote infection [67]. Processive movement of cargo by dynein normally requires the formation of a three-member protein complex composed of the dynein motor, dynactin activator, and a cargo adaptor which confers cargo specificity [68,69,70]. However, whether the force generated by this three-member protein complex is needed to disassemble SV40, or if an individual component of this complex acts to disassemble the virus, remains unclear. It is interesting to note that dynein-mediated disassembly of SV40 is reminiscent of the kinesin-1-dependent uncoating of adenovirus before entry of the virus into the nucleus [71]. These similarities underscore the importance of cellular motors during entrance of nonenveloped viruses into the nucleus. In addition to dynein, a cytosolic Hsc70-mediated chaperone system can also promote disassembly of SV40 [49,50,58,67] and the murine PyV [72]. Thus, it is in principle possible that this chaperone system operates in concert with dynein to disassemble PyV in the cytosol, priming the virus for nuclear entry.

SV40 uses the NLS located within its VP2 and VP3 proteins to engage importin α to gain nuclear entry [61,73]; whether the NLS of VP1 is involved remains unclear. For the BK PyV, the NLS of its VP2 and VP3 has been implicated in nuclear entry [74], and for the JC PyV, the NLS of its VP1 supports entry into the nucleus [74,75]. The murine PyV might use the NLS of its VP1, VP2, and VP3 to gain nuclear entry [76]. Thus, there are some variations amongst the PyV family in the use of NLS present within their viral structural proteins during this last viral entry step. Because the NLS of VP2 and VP3 are hidden in the native virion, conformational changes must be imparted to the virus to expose this region. This could take place within the ER where VP2 and VP3 are first exposed [43,56,77], or potentially when the virus reaches the cytosol where dynein and/or the Hsc70 chaperone system disassemble the virus [49,67].

An unaddressed question is the nature of the subviral particle that enters the nucleus. There is evidence to support the model that at least the VP3 of SV40 accompanies its genome into the nucleus [78,79], while VP1 of JC PyV reaches the nucleoplasm with its genome [74,75]. Aside from these data, there are very few additional insights into the viral components that arrive into the nucleoplasm. When and how the viral genome is ultimately uncoupled from its structural proteins is of obvious interest, as this information will undoubtedly inform critical nuclear events that initiate PyV infection. 

## 6. Conclusions and Future Directions

By far the most unique feature of productive SV40 entry is transport of the virus to the ER where subsequent penetration across the ER membrane enables viral escape into the cytosol. The only other virus reported to be trafficked to the ER is the HPV [80], although it is unclear whether transit to the ER represents the productive pathway for this virus. An often-asked question is why SV40 and other members of the PyV family target to the ER to cause infection. Part of the answer is that for SV40 to reach the cytosol, it must undergo conformational changes that expose its hydrophobic VP2 and VP3 proteins, generating a hydrophobic particle that can bind to and insert into a host membrane—this reaction initiates membrane penetration and escape into the cytosol. 

For these conformational changes to take place, forces that normally stabilize the overall architecture of the virus must be disrupted. Two dominant forces that support SV40 architectural integrity are the presence of a network of covalent disulfide bonds in the major structural protein VP1, as well as non-covalent associations of the C-terminal arms of VP1 that stabilize inter-pentamer interactions [44,45]. Importantly, as the ER is the only cellular organelle equipped with enzymes and chaperones that can disrupt both these covalent and non-covalent interactions [44,45], it reasons that SV40 must reach the ER and penetrate the ER membrane in order to access the cytosol.

Although a strict requirement for SV40’s transit through the ER during productive infection is clear, what remains unclear is the molecular basis by which SV40 reaches the ER and exits this compartment. For instance, future work must address how SV40 undergoes endosome-to-ER transport that bypasses the Golgi. Another critical question is how this viral particle creates a membrane penetration site (called foci) on the ER membrane via activation of the host motor kinesin-1 activity. The ability to construct its own membrane penetration portal is indeed a distinctive characteristic of SV40 entry. Finally, a fertile area of investigation is to clarify the last step of SV40 infection, namely, nuclear entry. To date, how SV40 is disassembled and delivered into the nucleus, and the nature of the viral components that enter the nucleoplasm, remain enigmatic.

Because SV40 displays similarities to human PyVs, insights from the study of SV40 entry will undoubtedly have major implications for human PyV infection. As human PyVs cause devastating diseases, clarifying the molecular basis of SV40 entry should provide new therapeutic strategies to combat PyV-related human diseases. Lastly, beyond the field of virology, elucidating SV40 infection should reveal basic cellular mechanisms, including membrane trafficking, transport, and penetration, as well as motor-cargo regulation.
